# Regulating Endogenous Neural Stem Cell Activation to Promote Spinal Cord Injury Repair

**DOI:** 10.3390/cells11050846

**Published:** 2022-03-01

**Authors:** Emily A. B. Gilbert, Nishanth Lakshman, Kylie S. K. Lau, Cindi M. Morshead

**Affiliations:** 1 Department of Surgery, Division of Anatomy, University of Toronto, Toronto, ON M5S1A8, Canada; emilyab.gilbert@utoronto.ca (E.A.B.G.); nishanth.lakshman@mail.utoronto.ca (N.L.); kylie.lau@mail.utoronto.ca (K.S.K.L.); 2 Institute of Medical Sciences, University of Toronto, Toronto, ON M5S1A8, Canada; 3 Institute of Biomedical Engineering, University of Toronto, Toronto, ON M5S3E1, Canada; 4 Donnelly Centre for Cellular and Biomolecular Research, University of Toronto, Toronto, ON M5S3E1, Canada

**Keywords:** spinal cord injury, spinal cord niche, neural stem cells, heterogeneity, neural repair, proliferation kinetics, microenvironment, migration, differentiation

## Abstract

Spinal cord injury (SCI) affects millions of individuals worldwide. Currently, there is no cure, and treatment options to promote neural recovery are limited. An innovative approach to improve outcomes following SCI involves the recruitment of endogenous populations of neural stem cells (NSCs). NSCs can be isolated from the neuroaxis of the central nervous system (CNS), with brain and spinal cord populations sharing common characteristics (as well as regionally distinct phenotypes). Within the spinal cord, a number of NSC sub-populations have been identified which display unique protein expression profiles and proliferation kinetics. Collectively, the potential for NSCs to impact regenerative medicine strategies hinges on their cardinal properties, including self-renewal and multipotency (the ability to generate de novo neurons, astrocytes, and oligodendrocytes). Accordingly, endogenous NSCs could be harnessed to replace lost cells and promote structural repair following SCI. While studies exploring the efficacy of this approach continue to suggest its potential, many questions remain including those related to heterogeneity within the NSC pool, the interaction of NSCs with their environment, and the identification of factors that can enhance their response. We discuss the current state of knowledge regarding populations of endogenous spinal cord NSCs, their niche, and the factors that regulate their behavior. In an attempt to move towards the goal of enhancing neural repair, we highlight approaches that promote NSC activation following injury including the modulation of the microenvironment and parenchymal cells, pharmaceuticals, and applied electrical stimulation.

## 1. Spinal Cord Injury

Spinal cord injury (SCI) in mammals is a debilitating condition which leads to a spectrum of sensory and motor deficits. To date there is no cure for this condition. Deficits that occur due to SCI include those caused by the initial mechanical damage which results in damage to axons, breakdown of the blood-spinal cord barrier, as well as a cascade of secondary events that worsen the extent of injury [1,2,3,4]. Secondary events following SCI include vascular damage, inflammation, excitotoxicity, cell death, and the activation of astrocytes [2,5,6,7]. Combined, these events result in the infiltration of circulatory factors and cellular contents into the spinal cord [8,9]. Blood-borne macrophages enter the spinal cord and resident microglia react to the injury by releasing cytokines and chemokines (such as IL-1β, IL-6, and TNF-α) resulting in a rapid inflammatory response in the first several weeks following SCI [6,10,11]. Additionally, myelin associated factors such as myelin oligodendrocyte glycoprotein (MOG), myelin-associated glycoprotein (MAG), and myelin basic protein (MBP) are released into the cellular milieu [12,13]. Concurrently, neurotoxicity results from the release of excitatory neurotransmitters exacerbating neuronal and oligodendrocyte cell death through necrosis, and later apoptosis [5]. Oligodendrocyte precursor cells (OPCs), which comprise 5–10% of the cells in the spinal cord parenchyma, are activated and undergo morphological changes and increased proliferation at early times post-SCI [14,15].

The secondary injury following SCI leads to edema, chronic demyelination, and the formation of a glial scar which is composed of multiple cell populations including astrocytes, OPCs and fibroblast-like cells [16,17,18,19]. Within the glial scar, resident astrocytes are activated and proliferate, hypertrophy, and migrate to the site of injury [20,21,22,23]. The glial scar serves both positive and negative functions following SCI. While it limits the spread of injury, it also restricts structural repair of the spinal cord [16,17,19] Following injury, reactive astrocytes upregulate several genes including *Nes*, *Axin2*, and matrix metalloproteinase genes (*Mmp2*, *Mmp13*), relative to naïve astrocytes. Subsequently, reactive astrocytes become scar-forming astrocytes within the glial scar and express a different set of genes including *Cdh2*, *Sox9*, and *Slit2*, among others. In line with the appearance of distinct subsets of astrocytes following injury, recent work suggests that sub-populations of astrocytes may also be responsible for enhancing neurite outgrowth from parenchymal neurons following SCI, highlighting the dual role of the glial scar [19,24,25,26,27]. Schwann cells, the myelinating cells in the peripheral nervous system (PNS), have been shown to migrate into the CNS from the PNS in response to injury where they contribute to the glial scar and may play a role in remyelination [28]. Finally, in response to SCI it has been shown that NSCs and their progeny (together termed neural precursor cells, NPCs) in the periventricular zone (PVZ) surrounding the central canal within the spinal cord proliferate, migrate to the site of injury, and differentiate into mature neural cells [29,30]. Hence, while NSCs in the mammalian spinal cord are responsive to injury, their activation is insufficient for structural repair or functional recovery following SCI [30,31,32]. 

Notably, in regenerative competent species, a similar sequence of cellular events results in strikingly different outcomes, with structural repair and functional recovery observed across multiple groups including teleost fish, urodeles and some lizards [33,34,35]. Interestingly, the hallmark feature that is best correlated with spinal cord repair and fully recovered function, is the activation of endogenous spinal cord NSCs. In regeneration-competent species, NSCs are rapidly recruited to the site of injury where they give rise to differentiated cells that integrate into the newly formed tissue, underscoring the potential of stem cell-mediated regeneration in the CNS [34,35,36,37]. In the hunt for strategies aimed at harnessing endogenous mammalian NSCs for repair, clues can be found by (1) focusing future studies on understanding the mechanisms of spinal cord development, with the goal of recapitulation following injury [38,39,40] and/or (2) studying the properties of regenerative competent species to understand the cell and microenvironmental factors that facilitate NSC activation and genuine CNS regeneration [33,39,41,42]. Towards the goal of facilitating neural repair in mammals endogenous NSCs represent a promising target due to their potential to replace lost or damaged cells and re-establish the neural networks in the spinal cord. Here, we will examine distinct NSC populations found in the adult spinal cord, explore their lineage dynamics and kinetics following injury and their response to external cues [43].

## 2. Endogenous Neural Stem Cells

NSCs are rare, slowly dividing cells found along the entire neuraxis of the developing and mature CNS [44,45,46,47,48] and demonstrate two key stem cell properties: self-renewal and multipotentiality. These properties are demonstrated using the in vitro “neurosphere” assay [45,49,50]. The neurosphere assay is a robust and powerful approach to isolate and study the properties of NSCs and their progeny. In vivo, NSCs derived from the PVZ in the spinal cord do not display multipotency but instead, their progeny are aneurogenic under homeostatic conditions [51,52]. This aneurogenic phenotype is related to the microenvironment within the spinal cord. This was eloquently shown with cell transplant experiments where spinal cord derived NPCs gave rise to neurons following injection into the hippocampus, a neurogenic region of the adult forebrain [53]. These findings reveal the multipotency of spinal cord NSCs that is masked in the spinal cord milieu [54,55]. The aneurogenic phenotype of spinal cord NSCs is also seen following SCI, where activated NSCs exclusively generate gliogenic progeny, producing mostly astrocytes, which are found at the site of injury [30,56].

## 3. Adult Neural Stem Cells: A Heterogeneous Population of Cells

**Figure 1:**
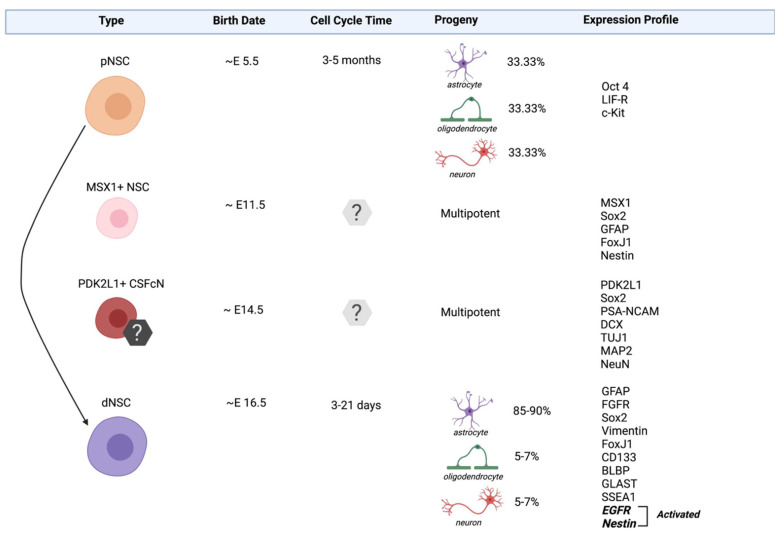
Neural stem cell populations in the spinal cord. Spinal cord derived cells that exhibit stem cell properties include primitive neural stem cells (pNSCs), MSX1+ NSCs, PDK2L1+ CSFcNs and definitive neural stem cells (dNSCs). The lineage relationship (solid arrow) is established between pNSC and dNSC populations. The lineage relationship of MSX1+ NSCs and PDK2L1+ CSFcNs is unknown. The expression profiles listed include genes known to be specific to the identities of a given population (e.g., Oct4, MSX1, and PDK2L1) and those related to their stemness.

NSCs within the mammalian spinal cord can be sub-divided into several functionally and molecularly distinct populations. A number of NSC populations can be isolated from the PVZ surrounding the central canal (Figure 1). Common to all NSCs is their ability to generate clonally derived, multipotent colonies of cells in vitro [48]. Similar to NSCs found in the brain, the majority of NSCs in the spinal cord are definitive NSCs [57], while a rare population of primitive NSCs have also been identified [48]. Most recently, single-cell sequencing (scRNA-seq) and proteomics, have facilitated a comprehensive profile of PVZ heterogeneity and identified a novel, MSX1+ population of NSCs [58,59]. Finally, recent work has demonstrated that NSC properties are elicited from a population of spinal cord niche cells known as cerebrospinal fluid contacting neurons (CSFcNs) in vitro. These cells have a neuronal phenotype in vivo yet in vitro, CSFcNs generate multipotent, self-renewing colonies, shedding light on a potential new source of NSCs [60].

Heterogeneity within the NSC pool is also defined on the basis of the stem cell “state” (i.e., quiescent versus activated). Most NSCs in the mammalian CNS exist in quiescence, a mitotically dormant state, without undergoing proliferation or differentiation [61]. Several physiological elements such as growth factors and physical exercise (for example) can cause quiescent NSCs to become activated to proliferate and generate NPCs [62,63]. Conversely, stress and old age are examples of factors that reduce proliferation and enhance NSC quiescence [62]. The ability of stem cells to switch between quiescence and proliferation has important implications for enhancing endogenous repair.

Increased resolution pertaining to the identification and potential of distinct NSC populations is fundamental to exploiting these populations for neural repair in mammals [59]. Here, we provide a current and more detailed description of the spinal cord NSC pools (Figure 1).

### 3.1. Definitive Neural Stem Cells (dNSCs)

Definitive neural stem cells (dNSCs) are the most abundant NSCs within the adult spinal cord. They are first identified on ~ embryonic (E)16.5 along the developing neuraxis and they persist into adulthood in the PVZ. dNSCs primarily exist in a quiescent state [64,65,66,67] and are identified based on their expression of markers including the intermediate filament proteins Nestin, Vimentin and Glial Fibrillary Acidic Protein (GFAP) [68], as well as transcription factor, SRY-Box transcription factor 2 (Sox2) [69] and cilia marker, forkhead box J1 (FoxJ1) [70]. In vitro, dNSCs are responsive to fibroblast growth factor (FGF) and epidermal growth factor (EGF), and form clonally-derived, multipotent, and self-renewing neurospheres in these conditions [71]. Following injury, dNSCs are activated [72] to proliferate and generate progeny and migrate to the site of injury where they primarily give rise to glial cells [48,73]. Lineage tracking studies have revealed that ~95% of the progeny differentiate into astrocytes which contribute to the glial scar and ~2–5% differentiate into oligodendrocytes [29,32,74]. Considering their abundance, responsiveness to injury and their ability to migrate to the site of damage, dNSCS are important targets for neural repair.

### 3.2. Primitive Neural Stem Cells (pNSCs)

Primitive NSC (pNSCs) are first isolated from the developing nervous system at E5.5 [75] and they persist throughout embryonic development and into adulthood in the PVZ of the adult mammalian forebrain [76,77,78,79] and spinal cord [76,77]. In the CNS, it is estimated that pNSCs are 1000-fold less frequent than dNSCs [76,77,78,79]. pNSCs are identified based on their expression of low levels of the pluripotency marker organic cation/carnitine transporter 4 (Oct4) as well as leukemia inhibitory factor (LIF) receptor and tyrosine-protein kinase Kit receptor (cKit) [77,79]. In vitro, pNSCs can be cultured using the neurosphere assay in the presence of LIF to form multipotent, self-renewing colonies. Similar to dNSCs, pNSCs can be activated following SCI leading to an increase in the size of the pNSC pool at early times following injury [48]. Different from dNSCs, pNSCs do not migrate from the PVZ as LIF responsive neurospheres have not been isolated from the lesion site following SCI [48,76]. To date, lineage tracking of these rare pNSCs has been performed in the brain [74], but remains elusive in the spinal cord.

While pNSCs are significantly less abundant than dNSCs, this population has properties that could potentially augment endogenous neural repair and regeneration following SCI. Unlike the more prominent dNSCs that primarily give rise to astrocyte progeny following differentiation in vitro and in vivo, Oct4+ pNSCs from along the neuraxis generate equal numbers of neurons, oligodendrocytes, and astrocytes in vitro [48], potentially conferring a benefit for the replacement of lost cells following SCI. Also relevant is that fact that pNSCs are lineally related to the abundant dNSCs (Figure 1). Studies using transgenic mice and antimitotic agents to specifically deplete dNSCs have shown that pNSCs can be recruited to replace the lost dNSCs, as well as contribute to neurogenesis in the adult forebrain [77,78]. Whether neurons are derived directly from the pNSCs or whether progeny must first pass through a dNSC intermediate stage, is still unknown. What is clear, however, is that pNSCs are upstream of dNSCs and are responsive to injury through enhanced proliferation. Together, these findings support the conclusion that pNSC activation could serve as a novel approach to increase the numbers of endogenous NSCs available for neural repair.

### 3.3. MSX1+ NSCs

Msh Homeobox 1 (MSX1) expressing NSCs are novel, radial glia-like stem cells that were identified in the spinal cord using RNA profiling, immunohistochemistry, and transgenic mouse models [58]. A conditional MSX1-CreER^T2^ mouse crossed to a tdTomato reporter (MSX1-Cre:tdTom) was injected with tamoxifen on E11.5 and tdTomato+ cells were identified in vivo when tissue was examined at E13. MSX1+ NSCs are restricted to the dorsal region of the PVZ and share several molecular markers with dNSCs including Nestin, GFAP, Sox2 and FoxJ1 [58,59]. Further, MSX1+ cells express cRET (GDNF and NTN growth factor receptors) which are found on hematopoetic stem cell populations and Id4 which is highly enriched in quiescent NSCs from the forebrain [80]. Similar to pNSCs and dNSCs, MSX1+ NSCS are a largely quiescent population in vivo [58]. MSX1+ cells isolated from tamoxifen labeled MSX1-Cre:tdTom mice can proliferate to form td-Tom+ neurospheres that are multipotent upon differentiation, thereby eliciting a cardinal property of stem cells. Intriguingly, MSX1+ radial-like NSCs have been previously identified in regeneration-competent species, where they have been shown as crucial for the success of tail regeneration [81]. While the response of MSX1+ NSCs following SCI has not yet been explored in mammals, the observed properties of the MSX1+ NSCs support the possibility that they represent an intermediate population between pNSCs and dNSCs in the NSC lineage, making them candidates for activation and recruitment following injury.

### 3.4. Quiescent vs. Activated NSC States

In the forebrain, the baseline activation state of resident NSCs has been studied in detail [67,80,82,83] and dNSCs (the best characterized) are shown to exist in two states, namely quiescent (qNSCs) and activated (aNSCs). qNSCs have a radial glia-like morphology and a slow cell cycle time of ~3–21 days [67]. qNSCs play a fundamental role in maintaining the stem cell pool into adulthood via asymmetric divisions [84]. Adult forebrain qNSCs are maintained in their slow cycling state using bone morphogenetic protein (BMP) and Notch signaling [85,86,87]. Conversely, aNSCs are non-radial, have short processes and are rapidly dividing with a short cell cycle time of <24 h in the adult mouse [88,89]. aNSCs express EGFR, proliferation markers such as Ki67 and proliferating cell nuclear antigen (PCNA) [67,89,90] whereas qNSCs do not express EGFR but do express the Helix-Loop-Helix transcriptional regulator Id2 and the transcription factor Sox9 [89]. scRNA-seq experiments have also revealed that alongside qNSCs and aNSCs, a primed-quiescent transitionary stage exists between the two [80,91]. These “primed” NSCs showed decreased Notch and BMP signaling and an increase in lineage-specific transcription factors and protein synthesis (i.e., EGFR, cyclin-dependent kinase 2 (CDK2)) [80]. With regard to neural repair, modulating the activation state of forebrain NSCs by pulling them out of quiescence has been shown to result from injury alone, as well as following the administration of growth factors, cytokines and drugs [61,92,93,94,95,96] Indeed, brain repair and improved functional outcomes have been correlated with the activation of endogenous NSCs [91,97,98,99].

NSCs in the spinal cord have been less well characterized in terms of their quiescent and activated states. However, it is clear that the proliferative profile of endogenous NSCs in the spinal cord is significantly less than the forebrain under homeostatic conditions. Proliferation markers such as Ki67 only identify rare mitotically active cells in the spinal cord PVZ suggesting that the vast majority of resident NSCs are in a quiescent state. In the case of MSX1+/ID4+ NSCs, their already quiescent phenotype can be further enhanced (decreased cell proliferation) in the presence of BMP6 in vitro [58]. Hence, with a sound knowledge of the factors that regulate the activation state of NSCs, the spinal cord NSC subpopulations could be recruited to contribute to SCI repair as has been demonstrated in the brain.

## 4. The NSC Niche

Understanding stem cell-intrinsic differences and lineage dynamics are fundamental to enhancing the potential of endogenous NSC populations following SCI, combined with the knowledge that NSC behavior is tightly linked to the environment, or “niche”, where they reside [55,100,101,102]. The stem cell niche plays a critical role in stem cell activation, proliferation kinetics and cell fate through cell-cell interactions including the release of secreted factors and cell-contact mediated interactions with neighboring cells [103], as well as cell adhesion [104]. In the CNS, factors such as age and sex also play important roles in NSC behavior [105]. For example, the aged niche is less capable of supporting stem survival and/or activation, which has important implications for stem cell-based therapies to treat the aged population. Hence, the microenvironment surrounding endogenous NSCs may be a key limiting factor in the realization of stem cell mediated neural repair [106,107].

**Figure 2:**
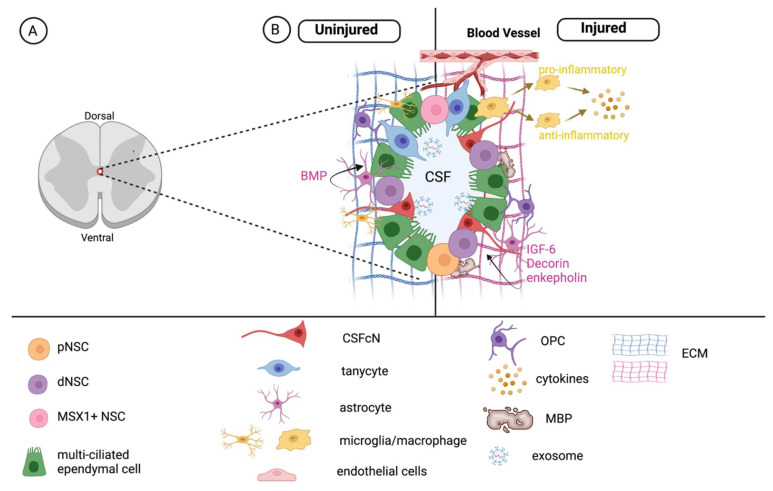
The NSC niche in the spinal cord. (**A**) Location of the central canal and PVZ. (**B**) The PVZ is composed of stem cells, multi-ciliated ependymal cells, cerebrospinal fluid contacting cells (CSFcNs), and tanycytes and is influenced by niche-associated astrocytes, oligodendrocyte progenitor cells (OPCs), microglia/macrophages, matrix components and exosomes. Each of these factors regulate NSCs in the uninjured (**left**) spinal cord. Following injury (**right**), the microenvironment is modified and leads to changes in the NSC pool.

The PVZ surrounding the central canal, including immediately adjacent parenchyma cells, comprises the spinal cord niche (Figure 2). The PVZ in the spinal cord is synonymous with the well-characterized subventricular zone (SVZ) of the brain in terms of developmental origin. However, the cellular architecture and molecular signaling within the spinal cord is less well characterized [29,58,100,108]. Notably, many morphologically and functionally distinct cells within the spinal cord niche share similar marker expression. As such, it is important to look beyond marker expression and examine cell behavior when identifying stem cell populations, including the fundamental properties of self-renewal and multipotency. Important recent work has begun to unravel the distinct characteristics in the mammalian (including human) spinal cord niche [58,59].

### 4.1. Ependymal Cells: A Population of NSCs?

Ciliated ependymal cells lining the central canal, identified by their expression of CD133 (Prominin1), FoxJ1 and Sox2 [109,110,111], play an important role in the circulation of cerebrospinal fluid [112]. Ciliated gene expression is affected by SCI (including expression of FOXJ1) and culture conditions, including the neurosphere assay [59]. Interestingly, ependymal cells have been ascribed as a source of multipotent NSCs that are activated following injury or following in vitro isolation [29,30,113]. In this regard, lineage tracking studies have reported varying results in terms of the potency and “stemness” of ependymal cells [29,30,31] Using transgenic mouse models that utilize the human promoter element to label FOXJ1 expressing cells, ependymal-derived cells were observed proliferating, migrating to the site of injury and differentiating into mature astrocytes and oligodendrocytes in models of SCI [29,30,32,73] Conversely, utilizing a different mouse model to label FOXJ1 ependymal cells (a knock-in model), ependymal cells were primarily unresponsive following SCI leading to the conclusion that ependymal cells are not NSCs in the mammalian spinal cord [31]. Whether the discrepancy between these studies can be entirely explained by the mouse model is not clear. Other considerations include the fact that ependymal cells can be further categorized based on ciliation patterns (uni-, bi-, and multi-ciliated) and there is a general lack of knowledge about the homology of these subpopulations in terms of gene and protein expression [114,115,116,117]. As a result, the stemness of ependymal cells continues to be an area of controversy in the literature.

If there are lessons to be learned from regenerative-competent species, they would support the potential “stemness” of at least a subpopulation of ependymal cells. Ependymo-radial glia (ERGs) are present in all species capable of spontaneous spinal cord regeneration and are defined based on their ciliation and location in the PVZ [37,55]. Structurally, ERGs have a long radial process that contacts the pial surface of the cord, similar to radial glia in developing mammals [36,41,118]. As with mammalian NSCs, ERGs express Nestin, Sox2, and GFAP [33,34,119]. Following injury, ERG activation (proliferation, migration, and differentiation) drives regeneration of the spinal cord and the re-establishment of regionally specific domains [33,34,36,37]. To date, comparative studies examining the homology of ERGs to distinct mammalian NSC populations remain unexplored. However, similarities in the morphology and protein expression of mammalian NSCs and ERGs highlight the idea that the lack of regenerative-competence in mammals may be related to a less-permissive niche.

### 4.2. Tanycytes—A Population of Glial Progenitors?

A sub-population of ependymal cells known as tanycytes are present in the spinal cord niche. Tanycytes are identified by their expression of Nestin, GFAP, Sox2 and Pax6 [114]. These cells have an apical process that reaches the cerebrospinal fluid (CSF) and a basal process that contacts the blood vessels [29,108,114,120]. Functionally, tanycytes filter molecules such as proteins and enzymes from the blood that normally cannot pass through the blood-spinal cord-barrier, and pass them into the CSF via transcellular transport [114]. Tanycytes have recently been proposed as a subpopulation of NSCs due to their ability to form self-renewing colonies following in vitro isolation. However, tanycyte derived colonies were not multipotent in vitro and were restricted in their differentiation to glial progeny (astrocytes, and oligodendrocytes) suggesting they are glial progenitors rather than bona fide NSCs [114].

### 4.3. Cerebrospinal Fluid (CSF) Contacting Cells: Immature Neurons, NSCs or Both?

Alongside ependymal cells, CSF contacting neurons (CSFcNs) are found within the PVZ [121]. CSFcNs can be subdivided into morphologically and functionally distinct Type I and Type II phenotypes. Type I cells express GABA, somatostatin and glutamate receptors and can fire action potentials [122,123]. Morphologically, CSFcNs have a bulb-like ending that project into the central canal and ventro-laterally oriented processes that project between cells [123,124]. Conversely, Type II CSFcNs have a flattened cell body and have thin lateral processes that can be directed dorsally, ventrally or laterally and do not show any active neuronal properties [123,125]. Common to both types of CSFcNs is the expression of PDK2L1 (TRPP2, a cation channel that localized to primary cilia) [126], along with canonical markers of neurons such as DCX, PSA-NCAM, and Nkx6.1 [121,127,128]. The function of Type II CSFcNs is thought to be related to sensing the composition and direction of flow of the CSF [121]. The ability of CSFcNs to regulate endogenous NSCs is not known. However, studies have shown that GABA release from CSFcNs inhibits NSC proliferation during development and in the hippocampus [129,130]. GABA could serve a similar function in the mature spinal cord.

Most interesting, very recent work has identified CFScNs as potential stem cell population in the adult spinal cord [60]. CSFcNs isolated via cell sorting for the pan-CSFcN marker PKD2L1 [124], specifically from the cervical spinal cord, were reported to generate multipotent colonies in the presence of growth factors used to isolate dNSCs (EGF and FGF2) [60]. Moreover, the CSFcN-derived colonies expressed dNSC markers (Sox2, GFAP and Nestin) and could be passaged multiple times [60]. This compelling stem cell behavior is unpredicted from cells that express a mature neuronal phenotype. While currently limited to in vitro characterization, important next steps to examine this population in vivo will determine whether CSFcNs are a novel subpopulation of spinal cord NSCs.

### 4.4. Endothelial Cells—Paracrine Modulators of NSCs

Endothelial cells that comprise blood vessels influence NSC kinetics including self-renewal and progenitor cell fate, through the release of paracrine factors including vascular endothelial growth factor (VEGF) and amyloid precursor protein (APP) [131,132,133]. Loss of function studies using interference RNA to reduce VEGF expression led to decreased Notch and Pten signaling and a concomitant reduction in NSCs and progeny resulting from reduced proliferation [132,134]. Additionally, using a transgenic conditional knockout mouse (TiE2-Cre:APP-floxed) to deplete APP production specifically from endothelial cells in the neurovascular niche, resulted in increased NSC proliferation in vivo. These findings highlight the role of endothelial cell-derived factors in regulating NSC activity [134].

### 4.5. The Extracellular Matrix: A Regulator of NSC Function

A number of extracellular matrix (ECM) proteins such as tenascin-C (TN-C), laminin, and chondroitin sulfate proteoglycans (CSPGs) can regulate NSC behavior in stem cell niches. TN-C is glycoprotein expressed by developing astrocytes in mammals that inhibits the proliferation of both NSCs and their progeny. This subclass of the tenascin family contains fibronectin and fibrinogen elements that, when knocked out in transgenic mouse models, lead to increased proliferation of NSC progeny as a result of increased FGF2 signaling [135,136]. Laminin, a prominent ECM protein found in basement membranes, has the opposite effect leading to enhanced NSC self-renewal and concomitant reduction in progenitor cell differentiation via the laminin receptor integrin α6β1 found on NSCs [137,138]. Other ECM molecules include CSPGs which are expressed by oligodendrocytes and secreted into the ECM. These have been shown to reduce the proliferation of NSCs in vitro and in vivo and this inhibition is lost when CSPGs are degraded through the application of the enzyme chondroitinase [104]. Hence, the impact of the ECM on NSCs and their progeny is clear and has important implications for neural repair as the ECM is markedly changed in disease and injury states.

## 5. Regulating Neural Precursors to Enhance Neurorepair

**Figure 3:**
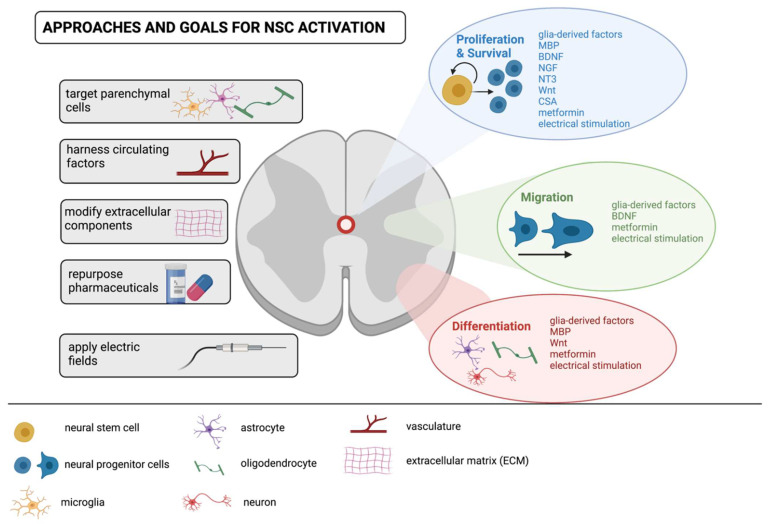
Approaches, goals and targets for modulating endogenous NSCs following spinal cord injury. Towards the goal of enhancing NSC activation to promote self-repair of the injured spinal cord, approaches encompass targeting the response of parenchymal cells to release NSC modulating factors (e.g., glia-derived factors, myelin basic protein (MBP), brain-derived neurotrophic factor (BDNF), nerve growth factor (NGF), neurotrophin-3 (NT-3)); modifying extracellular matrix components to regulate cell adhesion (for example),and/or administering pharmaceuticals (e.g., metformin and cyclosporin A (CsA)) that modify cell behavior through proliferation, survival, migration and differentiation. Novel therapeutics such as applied electric fields can also impact NSC behavior and is another approach that shows promise and warrants further investigation.

Improving structural repair and functional recovery following SCI in mammals remains an elusive challenge in the field of regenerative medicine. We hypothesize that improved outcomes will result from enhancing and modulating the proliferation, migration, and differentiation of endogenous NSCs and their progeny, as well as altering the niche to support, rather than inhibit, neurorepair (Figure 3). There are several promising approaches to improve the response of endogenous NSCs and alter the niche that surrounds them. Here, we describe parenchymal cell activity that impacts NSCs following injury. The promise of pharmacological agents to promote repair and the development of novel therapeutics, specifically the application of electrical stimulation, to mobilize NSCs following SCI.

### 5.1. Microglia, Astrocytes and Oligodendrocytes: Parenchymal “Influencers” on NSCs

Microglia, the immune cells of the CNS, are first responders following injury. Under homeostatic conditions microglia have a ramified morphology and sample the microenvironment for any perturbations in the spinal cord [139,140,141]. Following SCI, microglia and infiltrating macrophages, hereafter referred to as “microglia”, retract their processes and adopt an ameboid morphology in response to cytokines released from damaged cells in the parenchyma, such as interleukin 1ß (IL-1ß), tumor necrosis factor α (TNFα, interferon-γ (IFN-γ), adenosine triphosphate (ATP), and nitric oxide (NO) [140]. Activated microglia are proliferative cells that themselves release pro-inflammatory cytokines such as IL-1ß, IL-12, TNFα, and nitric oxide synthase (iNOS), which exacerbate the neurotoxicity [140,142,143]. Microglia can also be “alternatively” activated and release anti-inflammatory factors such as IL-10, transforming growth factor ß (TGFβ), and cluster differentiation 206 (CD206), which are neuroprotective [139,144]. Interestingly, microglia state is now well established as a continuum, rather than static states [145], hence modification of this population serves as possible avenue for altering NSCs. Irrespective of the activation state (pro- or anti-inflammatory), studies have shown that NSC behavior is modified in the presence of microglia. Using in vitro assays with microglia conditioned media, NSCs were less proliferative in the presence of pro-inflammatory microglia and generated more astrocytes relative to anti-inflammatory conditions. Interestingly, the migratory behavior of NPCs was increased in the Boyden chamber assay in the presence of anti-inflammatory microglia conditioned media [146,147]. Subsequent in vivo analyses revealed that the fate of NSC progeny is linked to microglia activation state whereby infusion of pro-inflammatory microglia-conditioned media drives a reduction in proliferation and neurogenesis, whereas anti-inflammatory microglia-conditioned media enhances NSC-derived neurogenesis and increases proliferation [146,147]. 

Extracellular-released vesicles such as exosomes provide a unique type of inter-cellular communication consisting of proteins, lipids, and nucleic acids. In the nervous system, microglia-derived exosomes (MDEs) contribute to neuron-glial crosstalk under homeostatic conditions as well as is in injury/diseased states [148,149]. Following injury, MDEs release factors including microRNAs (miRNAs) enriched in pro-inflammatory and neurotrophic signals. For example, MiR-155 MDE release has been shown to enhance pro-inflammatory microglia whereas MiR-21 can promote anti-inflammatory microglia [150,151,152]. Considering the ability of MDEs to release miRNAs, they may have a role in altering NSCs kinetics [149,152]. For example, MiR-21 has been shown to regulate EGFR and FGFR mediated signaling during neural development and could serve as a novel tool for modulating EGF- and FGF-responsive dNSC behavior.

In mammals, recent studies have revealed that activated microglia play an important role in limiting the injury following SCI [11,153]. Indeed, depletion of microglia after SCI disrupts glial scar formation and leads to reduced neuronal and oligodendrocyte survival. At the same time, it is well established that an extended pro-inflammatory state following injury can lead to increased impairments [140,142,154] Interestingly, in regeneration-competent species, pro-inflammatory cytokines in the early stages following injury are necessary for the success of NSC-mediated spinal cord repair. This was demonstrated using cytokine-specific zebrafish knock-out models whereby the loss of TNF-α and IL-1β impaired spinal cord regeneration. This study also revealed a notably shorter pro-inflammatory phase following injury in zebrafish [155]. These data support the hypothesis that shortened pro-inflammatory states that rapidly transition to anti-inflammatory states, are necessary for NSC-mediated spinal cord regeneration [155,156]. Hence, studies aimed at modulating the balance (both extent and duration) of microglia activation in mammals and examining the outcome on NSC activation are important next steps.

Alongside microglia, astrocytes are prominent effectors of the microenvironment [16,157]. Under homeostatic conditions, they provide support and protection to neurons [158] and after SCI, astrocytes are the principal cell type that contributes to the formation of the glial scar [16,19,25,159]. Astrocytes hypertrophy, proliferate, and migrate to the site of injury where they release pro and anti-inflammatory cytokines, chemokines and trophic factors [160,161,162]. There is considerable evidence demonstrating that reducing glial scar formation leads to worse structural and behavioral outcomes following SCI [11,32,163]. However, this is in contrast to what has been shown in regeneration competent species following SCI where there is a complete lack of glial scar formation [41,164,165,166]. To date, the mechanisms that underlie this scar-free healing and regeneration remain largely unexplored, but uncovering key molecules or astrocyte sub-populations present in species capable of spontaneous CNS repair would provide important insight into potential avenues to support NSC-mediated repair through glial scar modification. One hypothesis is that the proportion of neurotoxic (A1) and neuroprotective (A2) astrocytes [142,167] differs in species with varying regenerative capacity following SCI. Future studies exploring these differences in astrocytes and their effect on endogenous NSCs would be valuable to advance the field.

In mammals, it is known is that astrocytes influence the activity of endogenous NSCs, in the presence or absence of injury [168,169,170,171]. Within the spinal cord under homeostatic conditions, astrocytes release bone morphogenic protein (BMP) antagonists (i.e., Noggin) and differential screening-selected gene aberrative in neuroblastoma (DAN) to effectively limit the proliferation of NSCs and the differentiation of NSC progeny [100,172]. In contrast, specific to the brain, the secretion of BMPs promotes proliferation and differentiation of NSCs, leading to constitutive neurogenesis [173,174,175]. Further differences in astrocytes along the neuroaxis likely play a role regulating NSC behavior, with or without injury. For example, glutamate transporter 1 (GLT1) is responsible for taking up excess glutamate and protecting from excitotoxicity and is expressed 10-fold less in spinal cord astrocytes compared to those in the brain [176] Spinal cord astrocytes express higher levels of GFAP, as well as IL-6 and its signaling partner STAT3, which play a role in the injury response [177]. Further, regional distribution of the guidance molecule Sem3a is found in the spinal cord, with the highest concentrations ventrally, potentially impacting NSC behavior differentially within the PVZ [178].

Distinct gene profiles of astrocytes from neurogenic and non-neurogenic regions of the CNS have been identified along with a number of astrocyte-derived factors that inhibit NSC-derived neurogenesis (such as insulin-like growth factor-6, decorin and enkaphalin) [179]. Specific to the spinal cord, in vitro studies have used lipopolysaccharide (LPS) treatment to induce astrocyte activation and collected activated astrocyte-conditioned media (ACM) for NSC co-cultures [180,181]. These studies revealed significant increases in NSC proliferation and survival mediated by IL-6 in the ACM, as well as upregulation of the neurogenic transcription factors Mash1, Hes5, and Neurog1 [180]. With a sound knowledge of the astrocyte heterogeneity and astrocyte derived factors that regulate NSC behavior, both the activity of NSCs and their subsequent progeny could be regulated to promote neural regeneration.

Oligodendrocytes are the myelinating cells in the CNS. While the presence of mature oligodendrocytes has not been demonstrated to directly affect NSC behavior, exposure to oligodendrocyte-associated proteins released following injury/disease has been shown to have profound effects on their behavior [48,182] Following SCI, oligodendrocytes undergo rapid cell death which persists for up to three months post-injury [183]. One of the most abundant proteins comprising mature is myelin basic protein (MBP). Normally cytoplasmic is released into the microenvironment following SCI [184]. Both in vitro and in vivo studies have shown that MBP is inhibitory to NSC proliferation and NSC-derived oligodendrogenesis [48,182]. Most interesting, the observed inhibition is not a direct effect of MBP on NSCs, but instead is due to the indirect effect of MBP on spinal cord PVZ cells, which then release an unidentified factor that acts on NSCs [46,176]. Equally intriguing, this MBP-mediated inhibition of NSC activation and oligodendrogenesis is specific to PVZ cells from the spinal cord niche. The brain NSC niche does not release inhibitory factors as a result of MBP exposure [176]. With regard to NSC activation as a strategy to promote neural repair following SCI, sequestering released MBP or modulating its interaction with the niche may be an important future direction.

### 5.2. Bloodborne and Extracellular Factors

Cells within the PVZ are in direct contact with blood vessels [114] and numerous studies have revealed the impact of bloodborne factors on NPC behavior [185,186,187]. To date, most studies have focused on NSCs in the brain. However, these studies are useful for establishing key targets that could be applied, or reduced, to regulate NSCs in the spinal cord. Studies utilizing heterochronic parabiosis have shown that the infusion of TGF-β family member GDF11, a factor present in young blood, was sufficient to increase neurogenesis on aged mice [187]. Conversely, bloodborne factors corticosterone and chemokine CCL11 inhibit SVZ-derived neurogenesis [188]. Within the spinal cord, erythropoietin has been shown to enhance neurogenesis and oligodendrogenesis from NSCs isolated in vitro (although it’s efficacy and whether it has direct effects on NSCs in vivo following SCI is not clearly established) [189]. In addition, circulating growth hormones, as well as prolactin, regulate the proliferation and migration of endogenous NPCs in the brain [190,191,192].

Neurotrophic factor expression is altered following SCI [193,194,195]. While a multitude of neurotrophic factors have been shown to alter outcomes [193], we focus on those that have been shown to regulate NSC behaviors. Brain-derived neurotrophic factor (BDNF), nerve growth factor (NGF), and neurotrophin-3 (NT-3) are all upregulated following SCI and have all been shown to increase proliferation of endogenous NSCs in vitro [196,197,198]. Using in vitro assays, BDNF was shown to promote angiogenesis which drives NSC proliferation through the neurovascular niche [199]. The NSC neurovascular niche is distinct from non-neurogenic regions of the CNS and is characterized by interconnected, linear blood vessels that provide support for neuroblast migration as well as the delivery of bloodborne factors [200,201] The delivery of BDNF via transplanted NPCs genetically modified to over-express BDNF increased NSC survival (as compared to non-BDNF expressing NPCs), improved integration of NSC-derived progeny within neural circuits and promoted functional recovery [199,202]. The delivery of NGF increases NPC proliferation and survival and enhances the activity of injury induced neurotrophic factors such as NT-3 [203,204,205]. Infusing growth factors that are known to activate dNSCs, such as EGF and FGF2, also enhances NSC proliferation following SCI and promotes functional recovery [114,206,207]. Notably, the efficacy of NSC activating factors in promoting functional recovery has not been clearly demonstrated for the majority of factors.

Another approach for expanding the population of rare NSCs is through enhanced symmetric division. This would enable exponential expansion of the size of the NPC pool available for recruitment. Wnt proteins have been shown to play a role in symmetric cell division in several stem cell populations including hematopoietic, intestinal, and neural stem cells [208,209,210,211,212]. In the spinal cord, enhancing Wnt/ß-catenin signaling in NSCs via Wnt3a application both in vitro and in vivo, increased neuronal differentiation at the expense of astrocyte differentiation [213,214]. The pleiotropic effects of Wnt signaling in the CNS make it a promising factor to enhance neural repair via endogenous NPCs.

For all exogenous factor application strategies, defining the optimal timing, delivery method and location for delivery remain significant challenges for treating SCI. Most studies have applied exogenous factors in close proximity to the lesion and in the acute and subacute phases (i.e., within two weeks) following SCI [193]. Delivery of exogenous factors has been accomplished through direct injection, sustained release mini-pumps, transplantation of genetically modified cells, viral transduction directly into the parenchyma, as well as hydrogel or polymer-based delivery approaches. Each has demonstrated some degree of promise including tissue sparing, axonal outgrowth and/or functional recovery [193,215,216,217] but comparisons across studies are challenging due to the variations in experimental paradigms. Studies exploring the long-term impact of NSC activation are limited and could impact health outcomes [193,218] For instance, NSCs that are constitutively recruited into cycle post-injury may enter senescence, and senescent cells can negatively impact tissue health [219,220].

As with all interventions related to promoting neural repair, lessons from regeneration competent species may provide insight into establishing which neurotrophic factors, when and for how long, the exogenous factor should be delivered for optimal outcomes. For example, across teleost fish and urodeles, Wnt, EGF, FGF, BDNF, and NGF signaling have all been shown to play a role in the success of spontaneous, NSC-mediated, CNS regeneration [221,222,223,224,225]. In urodeles, EGF and FGF have been shown to be required for NSC proliferation and migration during spinal cord repair [226]. Studies exploiting the naturally occurring dynamics of these factors in spinal cord repair remains a useful, but under-utilized, resource when tasked with enhancing NSC-mediated repair.

### 5.3. The Pleiotropic Power of Repurposed Pharmaceuticals

The application of repurposed pharmacological agents is a particularly attractive approach for enhancing the response of endogenous NSCs, as it relates to their minimal invasiveness and the strong safety profile for drugs already in clinical use [95,227,228,229,230]. Examples repurposed drugs that have demonstrated effects directly on NPCs and/or niche cells that regulate their behavior include cyclosporin A (CsA) and metformin. CsA is a commonly used immunosuppressive drug to treat autoimmune disorders and to prevent graft rejection following organ transplant which has direct effects on NSCs [231,232]. Using both in vitro and in vivo assays, CsA prevented apoptotic cell death of NPCs through a calcineurin-independent inhibition of mitochondrial permeability pore formation. CsA expands the size of the NSC pool in both the brain and spinal cord [232,233]. At clinically relevant doses delivered systemically or intracranially through an intraventricular osmotic minipump or via a hydrogel composite, CsA was sufficient to promote functional recovery in two models of stroke (motor and cognitive) and in both rats and mice [234,235,236,237]. To date, its potential to enhance outcomes following SCI remains unexplored. Given CsA’s immunomodulatory properties, delivery at early times and for several days following SCI would have the dual role of dampening the immune response (mimicking what is observed in regeneration-competent species) and activating endogenous NSCs, providing a promising therapeutic strategy. Of note, CsA is commonly used in stem cell-based transplant studies to decrease graft rejection. Given the successful outcomes of many cell transplant-based interventions, it would be of interest to discern whether CsA’s effects on endogenous NPCs is also contributing the success of the therapeutic strategy.

Another repurposed drug that has shown considerable therapeutic promise for treating the injured CNS is the anti-diabetic drug metformin. Metformin has pleiotropic effects in the CNS. In NPCs, it stimulates adenosine monophosphate (AMP) kinase activity which activates the atypical protein kinase C (aPKC)-CBP pathway leading to increased proliferation and survival of NPCs through FOXO3 and Tap73 activity [228,229,238,239,240]. Furthermore, metformin has anti-inflammatory effects through AMPK-dependent and independent inhibition of NF-κB [241,242,243]. Metformin has also been shown to enhance glycolysis and promote angiogenesis [241,244,245,246].

Following brain injury, metformin leads to improved functional outcomes in pre-clinical animal models (stroke, traumatic brain injury and cranial radiation) and has led to promising results in children with acquired brain injury [98,99,240,247,248]. The positive outcomes are correlated with increased survival/proliferation of endogenous NSCs, NPC migration to sites of injury and enhanced neuronal and oligodendroglia differentiation [98,228,229], as well as with indirect effects through modification of the NSC niche by decreasing microglial activation following injury [247,249]. Metformin has demonstrated age and sex-dependent effects on brain NSCs—expanding the size of the NSC pool in adult females, but not adult males [248]. Additionally, metformin has been shown to influence OPCs following demyelinating injury, enhancing both their proliferation and differentiation into mature oligodendrocytes [250]. Indeed, CNS remyelination from OPCs after SCI has been attributed to resident spinal cord OPCS. There is some indication that newly formed OPC-derived Schwann cells may also play a role in remyelination [251]. Most relevant to SCI repair, recent work has shown that metformin delivery in the acute phase following SCI leads to improved functional outcomes in both males and females [239]. The pleiotropic effects of metformin make it challenging to discern the cell-based mechanism (s) that underlie the success of the treatment However, the strong correlation with improved functional outcomes and NPC activation suggest that endogenous NPCs, directly or through niche modulation, are playing a role in the positive outcomes.

### 5.4. Regulating Neural Stem Cell Behaviour with Electric Fields

Beyond pharmacological approaches, electrical stimulation has demonstrated promising evidence as a novel approach to activate NSCs towards the goal of facilitating neural repair. The significant role of electric fields (EF) as an environmental cue in development [252,253] and wound healing [254,255] shed light on its potential therapeutic application in neural regeneration. With an optimized stimulation paradigm, electrical stimulation following SCI could help to augment the intrinsic reparative response mediated by NSCs, enhancing their migration, and driving their differentiation into neurons and oligodendrocytes. In vitro and in vivo studies reveal that NSCs along the neuroaxis are electrosensitive cells, and their behavior can be regulated in terms of their kinetics, migration, and differentiation, by the presence of an EF [256,257,258]. Specifically, EFs have been shown to induce changes restricted to undifferentiated NSCs in a dose-dependent manner [257]. When stimulated, NSCs from both the brain and spinal cord undergo rapid, directed migration in vitro [257], and this has been elegantly demonstrated with spinal cord NSCs in organotypic slice cultures [259]. The EF application effectively guides NSCs to lesion sites where they can contribute to cell replacement, immunomodulation, and remodeling of damaged tissue [260]. Electric field application impacts a number of molecular and cellular signaling pathways including upregulation of phosphatidylinositol-3-kinase (PI3K) [259], Rac1/Tiam1/Pak-1 signaling pathway [261], and altering calcium influx dependent cell kinetics [262,263]. Applied EFs are potent enough to efficiently divert NSCs from their default migratory pathways [264], supporting their utility in directing NSCs towards the lesion site to aid in neural repair. Proliferation kinetics and differentiation of NSC progeny is also impacted by applied EFs which have demonstrated pro-survival effects on NSCs and enhance differentiation of progeny into neurons and oligodendrocytes [258] Recent studies have discovered the potential of EF in ameliorating neuroinflammation by shifting microglial states from pro-inflammatory to the anti-inflammatory phenotype at lesion sites in the CNS in vivo [265,266]. Taken together, EFs have the potential to enhance directed migration and increase the survival of NSCs post-injury through both direct effects and niche-mediated alterations making EF application a feasible and customizable approach to improve functional recovery following SCI. With optimized parameters, the ability of EFs to regulate NSC kinetic behavior affords a novel and exciting possibility to repair the injured spinal cord by harnessing the full potential of endogenous NSCs. More recently, clinically-focused therapies aimed to improve outcomes following SCI are investigating the application of epidural electric stimulation. To date, this has been proven to be a promising rehabilitation strategy when used in conjunction with physiotherapy [267,268]. The underlying cell-based mechanism is not well understood and studies investigating whether epidural stimulation alters endogenous NSCs following SCI would provide important insight regarding the mechanism by which motor function is restored.

## 6. Future Directions

Endogenous NSCs are promising cell target following SCI as a means to replace lost cells and potentially restore neural circuitry. It is unlikely that there is a single-bullet approach that will lead to SCI repair. Hence, combinatorial strategies that incorporate NSC activation and rehabilitation (for example) warrant further study. A comprehensive understanding of the NSC pool phenotypes, and factors that regulate their behavior, combined with a sound knowledge of which cells are most relevant for neural repair and improved function, are at the forefront of developing novel therapeutics. To this end, the field must continue to examine the contribution of endogenous NSCs to the regenerative process through foundational experiments such as lineage-tracking using viral vector and transgenic mice models. Novel approaches including NSC-subpopulation-specific ablation on neural repair will provide insight into the cell-based mechanisms that underlie cell replacement and/or functional recovery. One of the aspects of NPC-mediated repair that must be considered is the injury model, as maintaining the periventricular NSC niche is imperative for the proposed endogenous repair strategies. In considering different models of injury, the fact that dNSCs and pNSCs, specifically, are able to migrate within the parenchyma to get to the site of the lesion, suggests that neural repair mediated by NSCs “from afar” may still be a feasible approach. Beyond this, studies that explore the window of efficacy for NPC activation and sex and age-dependent factors that influence outcomes are also critical for realizing self-repair of the injured spinal cord.

## 7. Conclusions

The inherent capacity for NSC activation in mammals is promising in terms of harnessing the potential of NSCs for repair of the injured spinal cord. We propose that a comprehensive understanding of resident NSCs characteristics, their lineage relationship, and the factors that regulate their behavior will enhance our ability to unlock the regenerative potential of the mammalian spinal cord. While the complexity of the pathophysiology that follows SCI remains a challenge, it is also a relatively widespread feat that across the animal kingdom, many groups, including teleost fish, urodeles, and some lizards are capable of both perfect and imperfect structural repair of the spinal cord which results in functional recovery. What remains a challenge in the field is establishing whether improved structural and functional outcomes are mediated by endogenous NSC activation. Indeed, while correlations between NSC activation and improved outcomes have been demonstrated, it is unclear whether NSCs are necessary and/or sufficient for the beneficial results. Studies using lineage tracking and NSC ablation models are critical to answer this important question. Further, future studies should employ and exploit the regenerative expertise of these species to establish key targets, NSC sub-populations, and environmental factors that are required for successful spinal cord repair. Future goals include the identification of spinal cord NSC subpopulations and using lineage tracking to identify their contribution to neural repair following interventions that directly, or indirectly through the niche, enhance their activation in the injured spinal cord. Considerations of timing, degree of injury, as well as age and sex will undoubtedly impact structural and functional outcomes following SCI and are critical aspects to consider to ultimately realize endogenous NSC activation strategies to treat SCI survivors.
